# Artificial intelligence for detection of retinal toxicity in chloroquine and hydroxychloroquine therapy using multifocal electroretinogram waveforms

**DOI:** 10.1038/s41598-024-76943-4

**Published:** 2024-10-22

**Authors:** Mikhail Kulyabin, Jan Kremers, Vera Holbach, Andreas Maier, Cord Huchzermeyer

**Affiliations:** 1 Pattern Recognition Lab, Department of Computer Science, Friedrich-Alexander-Universität Erlangen-Nürnberg, Martensstr. 3, 91058 Erlangen, Germany; 2 Department of Ophthalmology, University Hospital Erlangen, Schwabachanlage 6, 91054 Erlangen, Germany

**Keywords:** Electroretinogram, Chloroquine, Maculopathy, Drug regulation, Retina

## Abstract

Chloroquine and hydroxychloroquine, while effective in rheumatology, pose risks of retinal toxicity, necessitating regular screening to prevent visual disability. The gold standard for screening includes retinal imaging and automated perimetry, with multifocal electroretinography (mfERG) being a recognized but less accessible method. This study explores the efficacy of Artificial Intelligence (AI) algorithms for detecting retinal damage in patients undergoing (hydroxy-)chloroquine therapy. We analyze the mfERG data, comparing the performance of AI models that utilize raw mfERG time-series signals against models using conventional waveform parameters. Our classification models aimed to identify maculopathy, and regression models were developed to predict perimetric sensitivity. The findings reveal that while regression models were more adept at predicting non-disease-related variation, AI-based models, particularly those utilizing full mfERG traces, demonstrated superior predictive power for disease-related changes compared to linear models. This indicates a significant potential to improve diagnostic capabilities, although the unbalanced nature of the dataset may limit some applications.

## Introduction

Electroretinography (ERG) allows objective evaluation of retinal function in vivo. It has been used in clinical and research settings for years and is now highly standardized. However, widespread use is limited by the need for dedicated equipment and, even more, by the difficulties in interpreting the results. Artificial Intelligence (AI) may, therefore, help to make this important technique more widely available in a clinical setting. Screening for toxic maculopathy using the multifocal electroretinogram (mfERG) is a good start for exploring the application of AI on ERG traces.

**Fig. 1:**
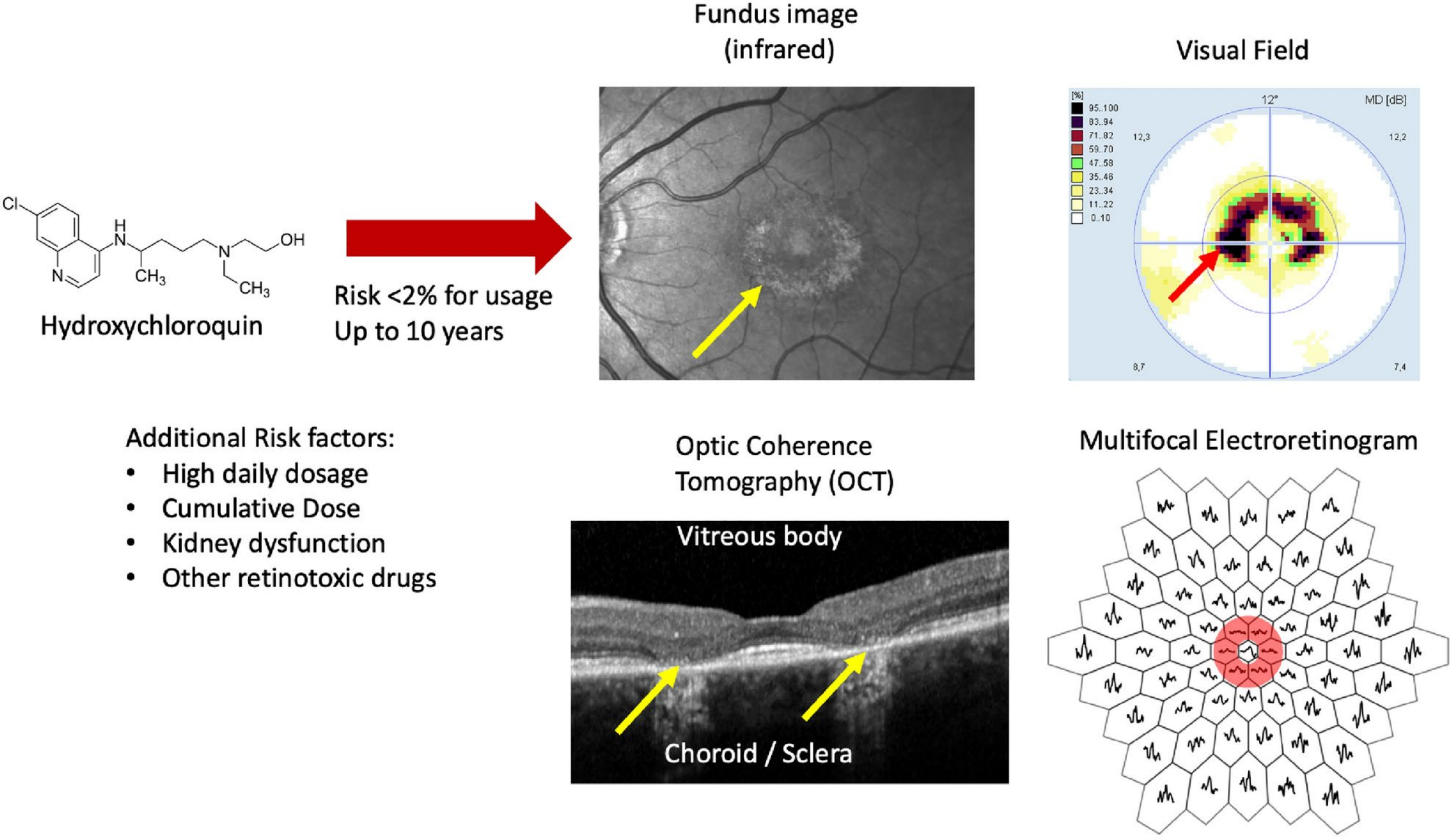
Clinical example of advanced toxic maculopathy. Hydroxychloroquine is commonly used for systemic lupus erythematosus (SLE) and other autoimmune diseases like rheumatoid arthritis, Sjögren’s syndrome, and lichen ruber planus. In contrast to other drugs used for the treatment of these diseases, hydroxychloroquine has few side effects and is well tolerated. The risk for toxic maculopathy increases with prolonged therapy but remains below 2% in the first ten years^1^. Therefore, “therapy should not be stopped casually for borderline findings”^1^. However, toxic maculopathy can lead to severe disability when not detected in time. The images shown here depict advanced disease with visible changes of the retina (Bull’s eye maculopathy, yellow arrow). The ocular coherence tomography section shows a clear loss of cells in the outer retina (where the photoreceptive cells are located) eccentric to the point of sharpest vision (yellow arrows). The perimetric examination demonstrates ring-shaped scotomata (red arrow), and the mfERG shows severely reduced amplitude in the paracentral segments (second ring, red circle). In even more advanced cases, central vision is also lost, leading to severe disability.

Chloroquine and hydroxychloroquine are important drugs in rheumatology. They are used for treating autoimmune disease because they are effective and generally much better tolerated than comparable drugs^1^. However, there is a risk of retinal toxicity, which may lead to considerable visual disability if the drug is not discontinued on time^1,2^. Figure 1 shows a clinical example.

The drug accumulates in the retinal pigment epithelium (RPE)^3^due to the high melanin content in these cells. Slow release from this reservoir prolongs exposure even after discontinuation of the drug. The mechanism of retinal toxicity is not completely understood. Intracellularly, chloroquin and hydroxychloroquine accumulate in the lysosomes. Damage to RPE cells but not to photoreceptors was demonstrated in vitro^4^. At some point in time, this accumulated drug may lead to cellular damage with changes in the outer retina and, subsequently, loss of pigment epithelium and choriocapillaris, leading to a characteristic Bull’s eye maculopathy. This process is not deterministic and requires regular screening.

Currently, the gold standard for screening is a combination of retinal imaging with Optical Coherence Tomography (OCT) and standard automated perimetry^2^. Using sophisticated imaging methods, subtle changes can be demonstrated in all patients under therapy^5,6^. Longitudinal studies show that progressive thinning of the retina precedes the onset of toxic maculopathy^7,8^.

ERG is an electrical response elicited by visual stimulation that is recorded from the eye. The ERG waveform can be used for the diagnosis of conditions affecting the retina, such as inherited or acquired diseases^9^. The mfERG technique allows the recording of local ERG responses simultaneously from many regions of the retina^10^ conventionally a hexagonal pattern. Individual hexagons are either black or white, following a pseudo-random (M-) sequence, allowing the extraction of local contributions to the ERG^11^. The mfERG is accepted as a screening tool for toxic maculopathy. However, special knowledge of retinal physiology is necessary for recording ERGs. Therefore, this technique is currently available only in large, specialized tertiary care centers^2^. Possibly, AI may help make this method more widely available.

To the best of our knowledge, a possible intermediate stage revealing functional damage in the absence of irreversible structural changes has not been clearly demonstrated, although researchers look for such early stages^5,12^. Such earlier stages could possibly be identified through a data-driven approach using machine learning (ML). Therefore, the current goal of screening for toxicity is demonstrating early functional and structural damage so that irreversible damage is avoided without discontinuing the drug too early owing to borderline findings^2^.

mfERG and perimetry are two sets of methods for assessing visual function when screening for toxic maculopathy. While the mfERG are responses recorded directly from the retina, perimetry assesses subjective detection thresholds of luminance modulation in selected areas of the visual field. This perception depends on retinal function, but also on post-retinal pathways, brain function and attention. Therefore, one would expect a systematic, albeit complex, relationship between the two paradigms.

Both methods need a thorough understanding of visual processing and are not always easy to interpret. Therefore, ML approaches might facilitate the application of these methods^13^. ML approaches are possibly able to identify and classify small changes that may be missed even by experienced clinicians (data-driven classification). However, available datasets are often too small, unbalanced, and incomplete. When larger datasets are pooled from different centers, problems can arise from inconsistent clinical standards (although the mfERG is highly standardized^10,11^) and differences between manufacturers of perimeters and ERGs.

We collected a real-world dataset from a tertiary care retina clinic that consisted of longitudinal mfERG and perimetric measurements from patients under therapy with chloroquine or hydroxychloroquine. Our main focus was to establish whether an AI algorithm based on the raw mfERG traces in the time-series domain outperforms a model based only on the clinically established readout parameters of the mfERG traces.

We were not able to look for early, pre-structural maculopathy because no patient showed a clear progression during follow-up. In order to identify promising approaches for further research, we trained models to identify clinically visible toxic maculopathy (classification task) and for predicting visual field sensitivities (regression task). When comparing with established readout parameters, we used a model trained only on ERG waveform parameters for classification and a linear regression model as a baseline for sensitivity prediction.

Although ML has been applied on full traces of full-field ERGs^13–16^and on selected parameters of mfERG^17^, this is, to our knowledge, the first study that used ML algorithms on full traces of mfERG data.

## Methods

### Patients

Data collection started by identifying patients from the ERG database who had undergone at least four mfERG examinations. From this cohort, we selected patients who had been examined to identify or screen for (Hydroxy-)chloroquine maculopathy. The patients in our retina clinic were predominantly Caucasian, but this was not a selection criterion. Demographic and basic clinical data were collected in a separate database. All patients screened for toxic maculopathy also underwent perimetric assessment of the central ($10^{\circ }$) visual field, OCT, and an examination of color vision.

### Instruments

All examinations were carried out according to our clinical procedure standards. For the critical outcome parameters, these standards were as follows:

White-on-white standard automated perimetry (SAP) measurements were performed with Octopus 900 perimeter (Haag-Streit, Köniz, Switzerland). Patients were positioned in front of the perimeter in a calm, darkened room. Corrective lenses were placed in front of the eye according to a table provided by the manufacturer. Background luminance was 10 $cd/m^2$. Goldman size III stimuli with a presentation time of 100 *ms* were presented in 81 loci covering the central field up to $10^{\circ }$ eccentricity (M pattern). Thresholds were determined with the “dynamic” algorithm. Results were exported as CSV files and further analyzed using the R statistical software and the visualFields package. Perimetric sensitivites were de-logarithmized for averaging of several locations within an mfERG hexagon.

mfERGs were recorded with the RETIscan system (Roland Consult, Brandenburg, Germany) using a hexagonal grid consisting of 61 hexagons. Pupils were dilated with Tropicamide and Phenylephrine eyedrops prior to examination. Under photopic conditons, the patient was positioned with his head in a chin rest. The distance to the monitor was 28 *cm*. Fiber electrodes were placed on the conjunctival fornix, reference electrodes were positioned laterally to the orbital rim, and ground electrodes were placed on the forehead after cleaning the skin with NewPrep. Impedance was $<10\,kOhm$. ERGs were recorded separately in the two eyes. Stimuli were presented on a CRT monitor. The stimulus consisted of 61 hexagonal segments, with larger hexagons toward the periphery (view angle $30^{\circ }$, distortion factor = 1:4). At least five cycles of the M-sequence were recorded, and recordings with artifacts were removed manually. The recordings were band-pass filtered with cutoff frequencies of 10 *Hz* and 100 *Hz*. Recordings were exported as CSV files and further analyzed using the R statistical software.

**Table 1:** Criteria for the OCT classification. Note that only the OCT information was used as a ground truth for the classification task.

Group	Criteria
Normal	Normal ellipsoide zone (EZ)
	Visible interdigitation zone (IZ)
	Normal outer nuclear layer (OPL)
	Normal inner plexiform layer (IPL)
Suspected toxic maculopathy	Disappearance of IZ
	Decreased reflectivity of EZ
Definite toxic maculopathy	Parafoveal thinning of ONL
	Parafoveal thinning of IPL
	Loss of EZ and/or RPE

**Fig. 2:**
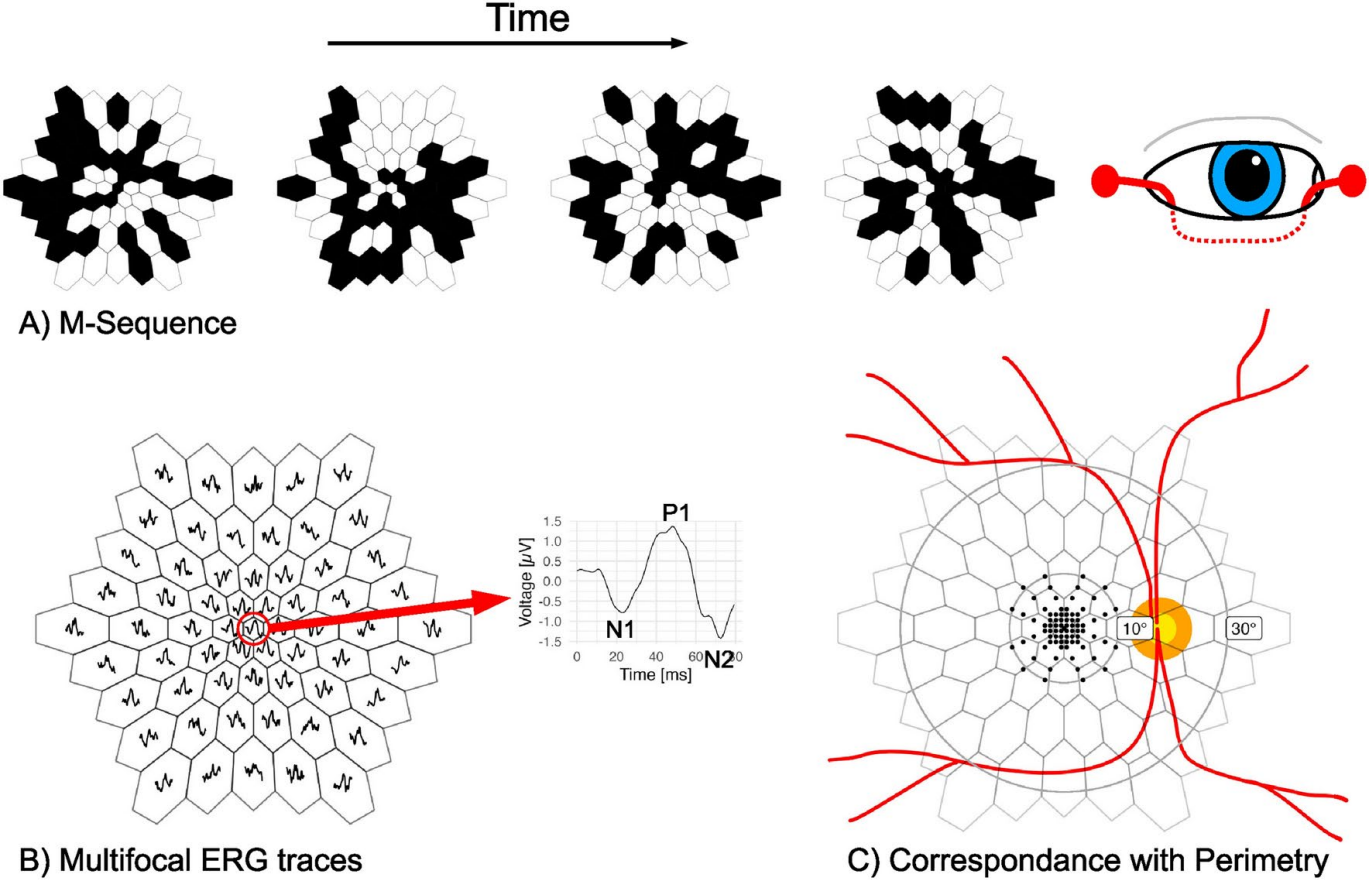
Multifocal ERGs. (**A**) The mfERG technique is based on a hexagonal grid stimulus with pseudo-random changes from black to white and vice versa in each hexagon. A sum potential is recorded with a fiber electrode that is placed on the conjunctiva. (**B**) The contribution from each hexagon can be extracted by averaging the time frames recorded signals at the times when the hexagon was white or black, respectively. This allows the extraction of the ERG response elicited by each hexagon. The responses are characterized by two troughs (N1 and N2) and one peak (P1). (**C**) The stimulus covers the central $30^{\circ }$ of the retina, which corresponds the posterior pole up to the large retinal vessels. The dots show the stimulus locations and sizes used for testing perimetric retinal sensitivity. The Octopus M pattern covers the central $10^{\circ }$.

OCT images were obtained with the Heidelberg Retina Analyzer (HRA2+OCT, Heidelberg Engineering, Heidelberg, Germany). At least one horizontal and one vertical scan covering $30^\circ$ of the posterior pole were obtained (ART 100 frames, 768 A-scans, 8.8/*sec*). These scans were qualitatively graded by one of the authors (CH), subdividing baseline findings into three groups: 1) normal OCT scans, 2) suspected toxic maculopathy, and 3) definite toxic maculopathy according to the criteria shown in Table 1. Changes in the inner retina were included in the OCT classification criteria because they are reported in the literature^18–20^. However, it has to be stated that there is some controversy about this^4^. In this study, no subject happened to be classified as definite maculopathy based solely on IPL changes. The presence of Bull’s eye maculopathy seen in the funduscopic examination due to atrophy of the retinal pigment epithelium is a sign of advanced disease (see Fig. 1). This sign was not used for classification because classification is based only on OCT criteria. Advanced disease was not an exclusion criterion for this study (see Limitations section).

### Data collection

As the raw mfERG and perimetry data were exported as CSV files, one sensitivity value [*dB*] was available for each locus in the visual field, and one mfERG trace was available for each hexagonal segment. A 3 *dB* decrease in perimetric sensitivity indicated that the stimulus had to be twice as luminant in order to be seen by the patient in the given location compared to a healthy subject. Furthermore, the readouts of the standard parameters of the mfERG traces determined by the software provided by the manufacturer were exported because they represented the standard of care. These parameters consisted of amplitudes and implicit times of the N1, P1, and N2 peaks/troughs of the mfERG traces. The spatial relationship and the standard parameters are illustrated in Fig. 2. We utilized the P1 amplitudes for the classification task because only one ring (ring 2) was used, but we employed the P1 amplitude densities (P1 amplitudes divided by hexagon areas in $deg^2$) for the regression task. Table 2 shows the distribution of the collected data.

**Table 2:** Dataset distribution.

	Norm	Maculopathy	Suspected
	**Subjects**		
	35	9	9
	**Used Traces**		
Left eye	12993	2745	3477
Right eye	13176	2745	3660
Total	26169	5490	7137

The study was approved by the ethics committee of the Medical Faculty of the Friedrich-Alexander University Erlangen-Nürnberg (23-379-Bm, 23 November 2023). The study adhered to the tenets of the Declaration of Helsinki. Informed written consent was obtained from all subjects involved in the study.

## Deep learning approach

Deep Learning (DL) methods allow the investigation of the entire ERG signal and not only the standard readout parameters as in the classical approach. Analyzing the complete signal offers significant advantages: it automatically allows the model to learn relevant features from raw time-series data. Time-series data can contain complex and hierarchical patterns. It is difficult to identify these patterns manually and develop readout parameters that quantify these patterns (feature engineering). By analyzing the entire signal, DL models can uncover complex temporal relationships that might be missed when using only the conventional waveform parameters, such as amplitudes and implicit times.

We used two different approaches to the problem. The algorithm was trained to predict either a categorial variable (group) for each patient (classification problem) or a continuous variable that is associated with disease severity (regression problem). Here, we used the perimetric sensitivity as a surrogate parameter.

For training, the algorithm was confronted with with a subset of the raw data and a corresponding “ground truth” (training set). The connections of a network of “neurons” are adjusted during the training process. For evaluation, the algorithm made predictions on another subset of the data without knowing the ground truth, and the predictions were compared with the latter.

### Classification problem

In the first experiment, we address the problem of classifying the participants into healthy subjects and patients with maculopathy (classification problem). The classical approach is to classify patients by the values of amplitudes and latency of N1- and P1-wave components of the ERG signal. We applied a Random Forest Classifier (RFC) trained on these wave parameters for the baseline. RFC operates as an ensemble, or group, of ML models that collectively work better than single models. It combines multiple decision trees-models that make predictions based on a series of questions and answers about data features to improve the overall accuracy of predictions. This method helps reduce overfitting, a common problem where a model performs well on training data but poorly on unseen data, by ensuring the model is robust and performs consistently across different datasets^21^.

We employed two DL algorithms suited for analyzing full ERG traces: (a) Bidirectional Long Short-Term Memory (BiLSTM) and (b) a Time Series Transformer (TST) due to their ability to capture both temporal dependencies and long-range patterns in the sequential data, enhancing the model’s capacity to discern complex patterns in time series signals. BiLSTM^22^is a type of recurrent neural network (RNN)^23^designed to capture and learn from temporal dependencies in sequential data, making it particularly effective for analyzing sequences with long-term relationships. TST^24^ is an advanced architecture based on transformer models, a newer class of neural networks originally developed for natural language processing. Transformers excel at handling sequential data by using “self-attention mechanisms,” which allow the model to weigh the importance of different points in the sequence. Due to them, TST effectively captures both short-term and long-term temporal dependencies and broader global patterns within the data, making it highly suitable for complex time series analysis.

#### Training

The training was performed independently for the second and third rings because they reflect the function of different retinal areas.

For the training and validation of the ML algorithms, we applied K-fold subject-wise cross-validation (K = 3): the dataset was split into three subsets, two of which were used for training and one for testing. Signals from one subject may appear only in one of the subsets. This procedure was repeated three times so that each subset was used for a test once. It was essential to ensure that patients with manifest maculopathy were present in both sets each time. Therefore, we applied stratified sampling.

During the training, oversampling was applied on the minority class every time for the training folds to avoid unbalancing (patients from the smaller groups could be sampled more than once). We used the CrossEntropyLossFlat loss function, designed to measure classification models’ performance by comparing the predicted probabilities against the actual class labels with a flattened output for improved optimization. This function was employed across all models with an added weight parameter to handle class imbalances. For optimization, we utilized the Adam optimizer, a widely used method that adjusts the learning rate for each parameter individually. The learning rates ranged between 0.0001 and 0.001, and accuracy was used as the primary metric to evaluate model performance during validation. The models were trained using data from both eyes. Validation of the algorithm was performed separately on the right and left eyes because the disease may affect the eyes symmetrically, and, therefore, measurements are not independent. For each patient, only the first mfERG experiment was taken into account. All models were trained on a local machine with AMD Ryzen 9 5900HX $\times 16$ processors and NVIDIA GeForce RTX 3070.

#### Metrics

For the evaluation of the classification problem, we computed the following metrics: Balanced Accuracy, Precision, Recall, and F1 score. These metrics provide a comprehensive classification evaluation by assessing its performance across different aspects, avoiding biases that can arise from relying on a single metric, especially in the imbalanced dataset. Balanced Accuracy offers an overall success rate. Precision and Recall become critical by focusing on the model’s ability to correctly identify patients with maculopathy without misclassifying healthy ones. The F1 score adjusts Precision and Recall, providing a metric that balances the importance of avoiding FP and FN.

1 $$\begin{aligned} & Precision = \frac{TP}{TP + FP}, \end{aligned}$$

2 $$\begin{aligned} & \quad Recall = \frac{TP}{TP + FN}, \end{aligned}$$

3 $$\begin{aligned} & \quad F1\, score = \frac{2 \times Precision \times Recall}{Precision + Recall}, \end{aligned}$$

where $$TP = True\ Positive$$, $$TN = True\ Negative$$, $$FP = False\ Positive$$, $$FN = False\ Negative$$.

4 $$\begin{aligned} Balanced\ Accuracy = \frac{Sensitivity + Specificity}{2}, \end{aligned}$$

where

5 $$\begin{aligned} & Sensitivity = \frac{TP}{TP + FN} , \end{aligned}$$

6 $$\begin{aligned} & \quad Specificity = \frac{TN}{TN + FP}. \end{aligned}$$

### Regression problem

In the second experiment, we investigated whether visual field sensitivities in the corresponding retinal location can be predicted from the mfERG traces. Predicting a continuous variable-sensitivity-based on known wave parameters is called a regression problem.

To solve the regression problem, we applied the classical Residual Network (ResNet) architecture, which was widely used for Electrocardiography (ECG) classification^25–28^and gated Multilayer Perceptron (gMLP)^29^- a novel approach to work with time-series data, that is an effective alternative to transformers^30^, that potentially allows the training convergence on smaller datasets and was recently applied to ERG^31^.

ResNet is a neural network architecture designed to address the vanishing gradient problem. This phenomenon occurs in the training of deep neural networks when the gradients used to update the network become extremely small^32^. ResNet was introduced by Kaiming He et al^33^. Skip connections in the network allow information to flow directly without being heavily processed. These connections enable the training of very deep networks. ResNet consists of residual blocks with multiple convolutional layers. Within a residual block, the input is added to the output of the block, effectively continuing the information. This helps in learning the residual functions, making it easier to optimize deep networks.

gMLP is a neural network architecture combining traditional elements of a Multilayer Perceptron (MLP) - a type of neural network composed of multiple layers of neurons, where each layer is fully connected to the next - with gating mechanisms inspired by Recurrent Neural Networks (RNNs) and transformers. Traditional MLPs have been widely used in various ML applications. However, they perform poorly on sequential data where the order of elements matters. On the other hand, RNNs and Transformers have been widely used for sequence modeling but have limitations: RNNs suffer from vanishing gradients, and transformers are computationally expensive.

#### Training

We performed a three-fold subject-wise cross-validation. We applied the same techniques for the training subsets to upsample the data related to the minority class (maculopathy) as for the classification problem. The models were trained using the entire dataset with ERG signals from both eyes.

We used “gMLP-Ti” - a tiny version of the model with a smaller model dimension (128) and a feed-forward dimension (768), which leads to a fewer number of trainable parameters and computational costs^29^. We set GELU as an activation function. Both models were trained until convergence with a maximum learning rate of 0.0001 and batch size of 32. All models were trained on a local machine with AMD Ryzen 9 5900HX $\times 16$ processors and NVIDIA GeForce RTX 3070.

**Table 3:** Demographic and clinical characteristics.

	Overall
	(N=53)
**Age at baseline (yrs)**	
Mean (SD)	51.8 (13.4)
Median [Min, Max]	54.8 [21.0, 83.4]
**Sex**	
Female	42 (79.2%)
Male	11 (20.8%)
**BCVA**^1^**: better eye (logMAR)**	
Mean (SD)	0.0389 (0.0625)
Median [Min, Max]	0.0179 [-0.0162, 0.301]
Missing	1 (1.9%)
**Mean defect: better eye (dB)**	
Mean (SD)	1.79 (1.92)
Median [Min, Max]	1.30 [-2.34, 6.80]
Missing	2 (3.8%)
**Diagnosis**	
SLE^2^	26 (49.1%)
RA^3^	8 (15.1%)
LRP^4^	3 (5.7%)
SS^5^	3 (5.7%)
Other	13 (24.5%)
**Medication**	
Chloroquine	10 (18.9%)
Hydroxychloroquine	37 (69.8%)
Both	6 (11.3%)
**Follow up (days)**	
Mean (SD)	1930 (1450)
Median [Min, Max]	1740 [0, 7650]

^1^Best-corrected visual acuity.

^2^Systemic lupus erythematosus.

^3^Rheumatoid arthritis.

^4^Lichen ruber planus.

^5^Sjögren Syndrome.

#### Metrics

Mean Absolute Error (MAE) and R-squared (R2) are commonly used metrics for evaluating regression models because they provide valuable insights into different aspects of model performance.

MAE represents the average absolute difference between the ground truth in the dataset and predicted values by a model. It measures the average of the residuals of the values:

7 $$\begin{aligned} MAE = \frac{1}{N} \sum _{i=1}^{N} \left| y_i - \hat{y}\right| , \end{aligned}$$

where $$\hat{y} - predicted\; value\; of\; y$$ and $$\overline{y} - mean\; value\; of\; y$$.

R2 is a statistical measure in a regression model that determines the proportion of variance in the dependent variable that can be explained by the independent variable. It shows how well the data fits the regression model:

8 $$\begin{aligned} R2 = 1 - \frac{SS_{res}}{SS_{tot}} , \end{aligned}$$

where $$SS_{res} - residual\; sum\; of\; squares$$ and $$SS_{tot} - total\; sum\; of\; squares$$.

## Results

### Demographic and clinical characteristics at baseline

Fifty-three patients, predominantly female, aged between 21 and 83.4 years (at baseline), were included in this study (see Table 3). These patients were monitored in periods lasting between 266 and 7650 days (0.7 - 20.9 years) with regular mfERG and perimetry examinations.

Thirty-five patients had no signs of toxic maculopathy (normal group), nine patients had minimal signs (suspects), and nine patients had manifest toxic maculopathy. No patient converted to another group during follow-up, so that no progression was detected.

### Classification problem

ROC analysis showed that ring 2 was the most potent diagnostic marker, followed by ring 3, and that the P1 amplitude was the best parameter for discriminating patients without maculopathy from those with manifest maculopathy. When averaging all measurements available for each eye, the area under the curve (AUC) for this P1 amplitude in ring 2 was 0.87 (0.86 for right eyes only, 0.89 for left eyes). According to Youden’s J statistic, the optimal cutoff was $0.45\,nV/deg^2$.

**Fig. 3:**
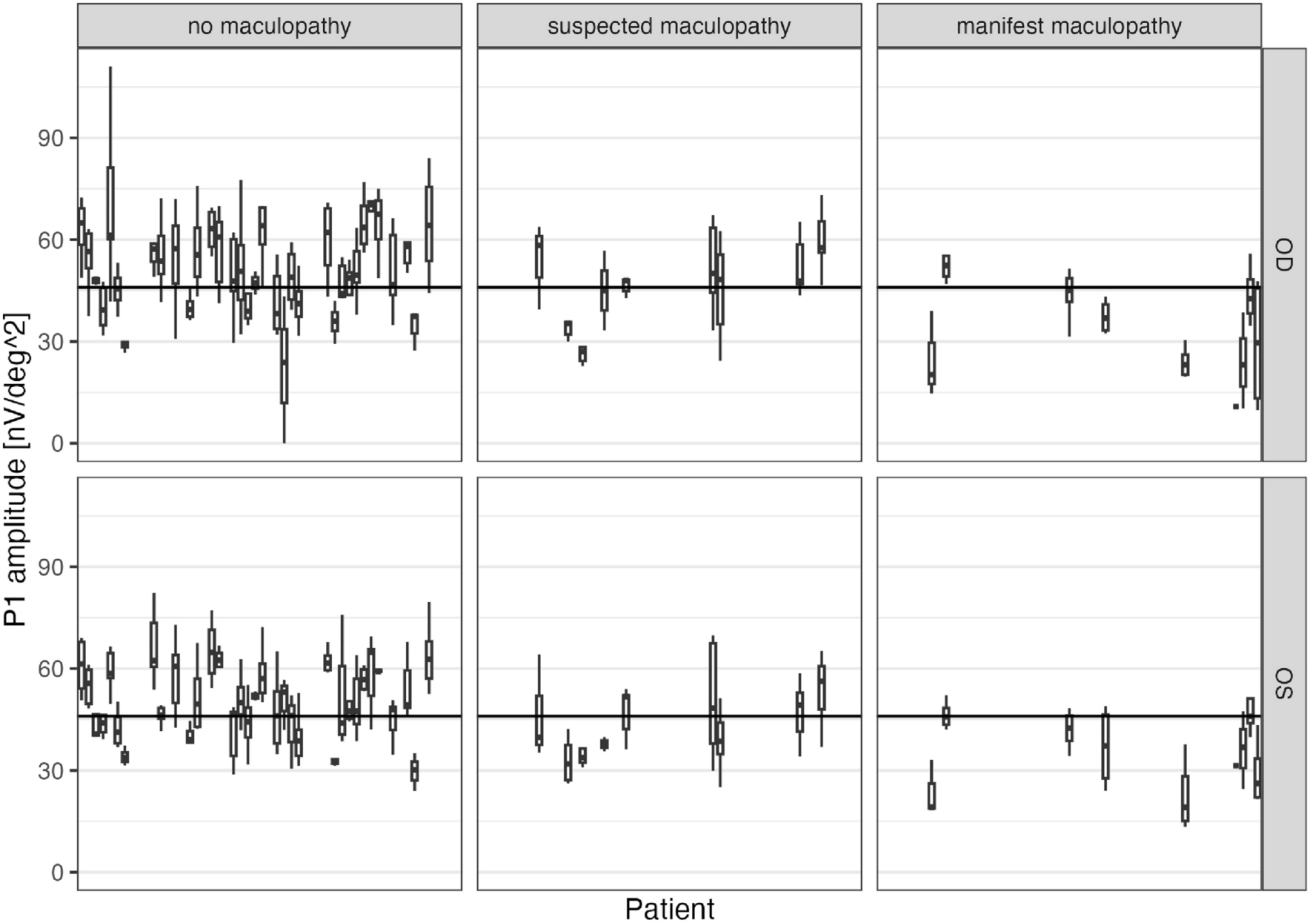
All amplitudes of all eyes grouped by clinical group and laterality. For each eye, measurements are summarized as boxplots. Note that marked intra-individual variability. The horizontal line represents the cutoff as determined by the Youden index in the ROC analysis comparing the patients without maculopathy and those with manifest maculopathy.

Figure 3 shows that intraindividual variability is relatively large compared to differences between groups, limiting diagnostic capability. The effect of the clinicial group on different ERG parameters ring 2 is shown in Table 4.

**Table 4:** Parameters Ring 2.

	No maculopathy	Suspected maculopathy	Manifest maculopathy
	(N=235)	(N=64)	(N=40)
**Peak time N1 (ms)**			
Mean (SD)	0.0242 (± 0.00239)	0.0244 (± 0.00270)	0.0235 (± 0.00371)
**Peak time P1 (ms)**			
Mean (SD)	0.0437 (± 0.00316)	0.0442 (± 0.00261)	0.0417 (± 0.00525)
**Amplitude N1 (nV/deg2)**			
Mean (SD)	20.3 (± 7.18)	17.2 (± 7.02)	15.0 (± 7.79)
**Amplitude P1 (nV/deg2)**			
Mean (SD)	52.6 (± 13.7)	48.1 (± 10.8)	37.1 (± 12.7)
**Amplitude 1st harmonic (nV)**			
Mean (SD)	25.1 (± 7.07)	23.0 (± 6.46)	17.4 (± 8.67)
**Mean Sensitivity (dB)**			
Mean (SD)	29.0 (± 1.97)	28.9 (± 1.86)	25.5 (± 4.08)

#### Evaluation of machine learning models

The results for the left and right eyes are shown in Table 5. The metrics show that the signals from ring 2 were superior for detecting toxic maculopathy. For the signals from both rings, the TST model performed best.

Note that the RFC is based only on the amplitudes. In contrast, BiLSTM and the TST models rely on the complete traces. This suggests that the traces contain additional information.

**Table 5:** Evaluation metrics of the models on the left and right eyes.

Model	Ring	Left eye				Right eye			
		Balanced Accuracy	Precision	Recall	F1	Balanced Accuracy	Precision	Recall	F1
RFC^1^	2	0.761	0.768	0.624	0.712	0.746	0.785	0.631	0.707
BiLSTM^2^	2	0.765	0.711	0.688	0.721	0.751	0.781	0.666	0.747
TST^3^	2	0.786	0.818	0.701	0.732	0.795	0.844	0.728	0.771
RFC	3	0.673	0.678	0.576	0.512	0.695	0.701	0.579	0.567
BiLSTM	3	0.694	0.751	0.583	0.656	0.708	0.733	0.625	0.717
TST	3	0.721	0.788	0.628	0.731	0.726	0.781	0.638	0.726

^1^Random Forest Classifier.

^2^Bidirectional Long Short-Term Memory.

^3^Time Series Transformer.

### Regression problem

**Fig. 4:**
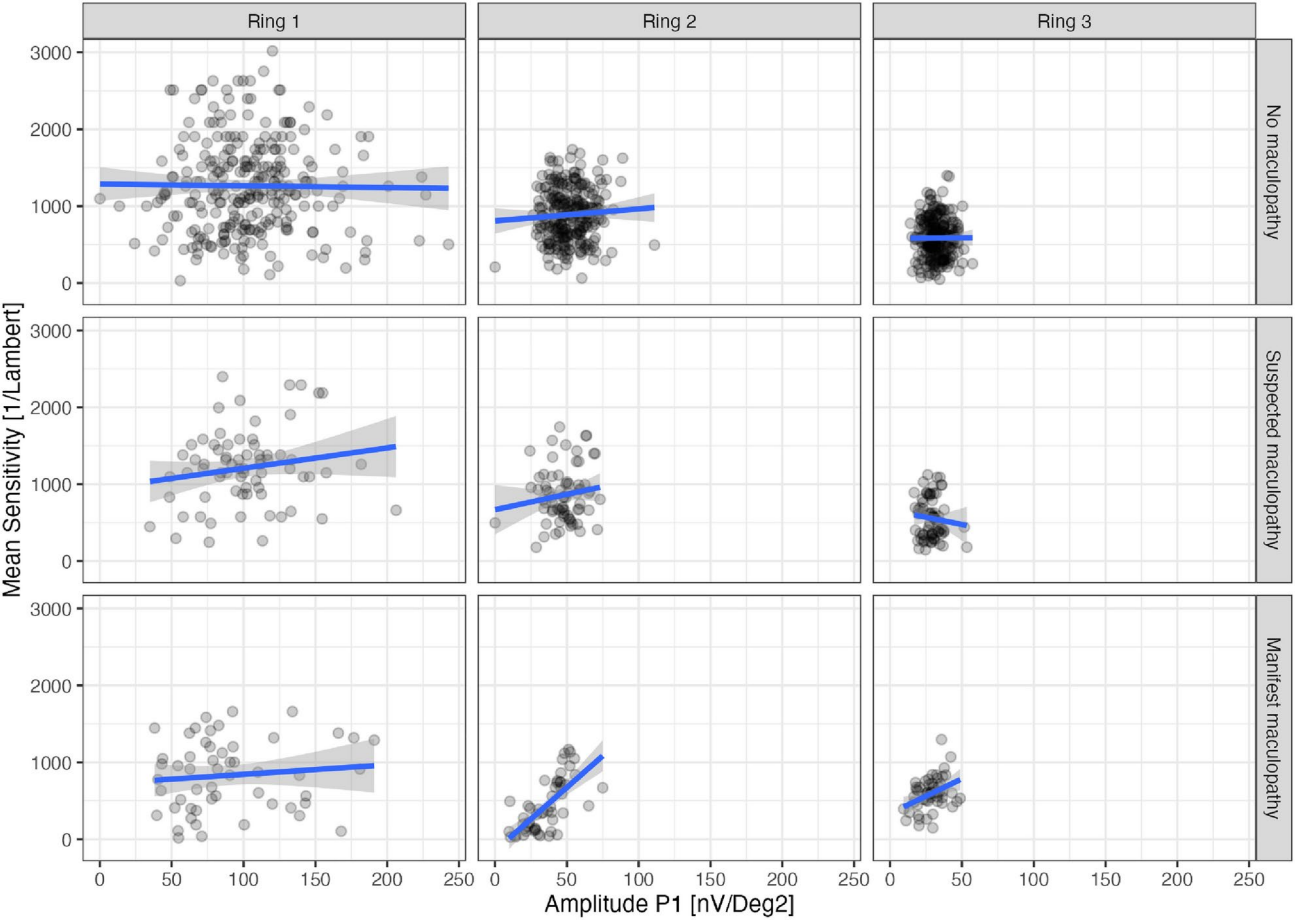
Correlation between mfERG amplitudes and perimetric mean sensitivity.

Figure 4 shows no apparent correlation between mfERG amplitudes and visual field sensitivities in patients without maculopathy (top row) and patients with suspected maculopathy (middle row). Please note that both mfERG amplitudes and mean sensitivities decrease with increasing eccentricity from ring 1 to ring 3. Ring 1 also displays the largest variability in mfERG amplitudes because it consists of only one segment and is not averaged.

In the patients with manifest maculopathy, there is a strong correlation between mfERG and mean perimetric sensitivity in ring 2 and a weaker correlation in ring 3 due to disease-related photoreceptor loss affecting both parameters.

#### Evaluation of machine learning models

**Fig. 5:**
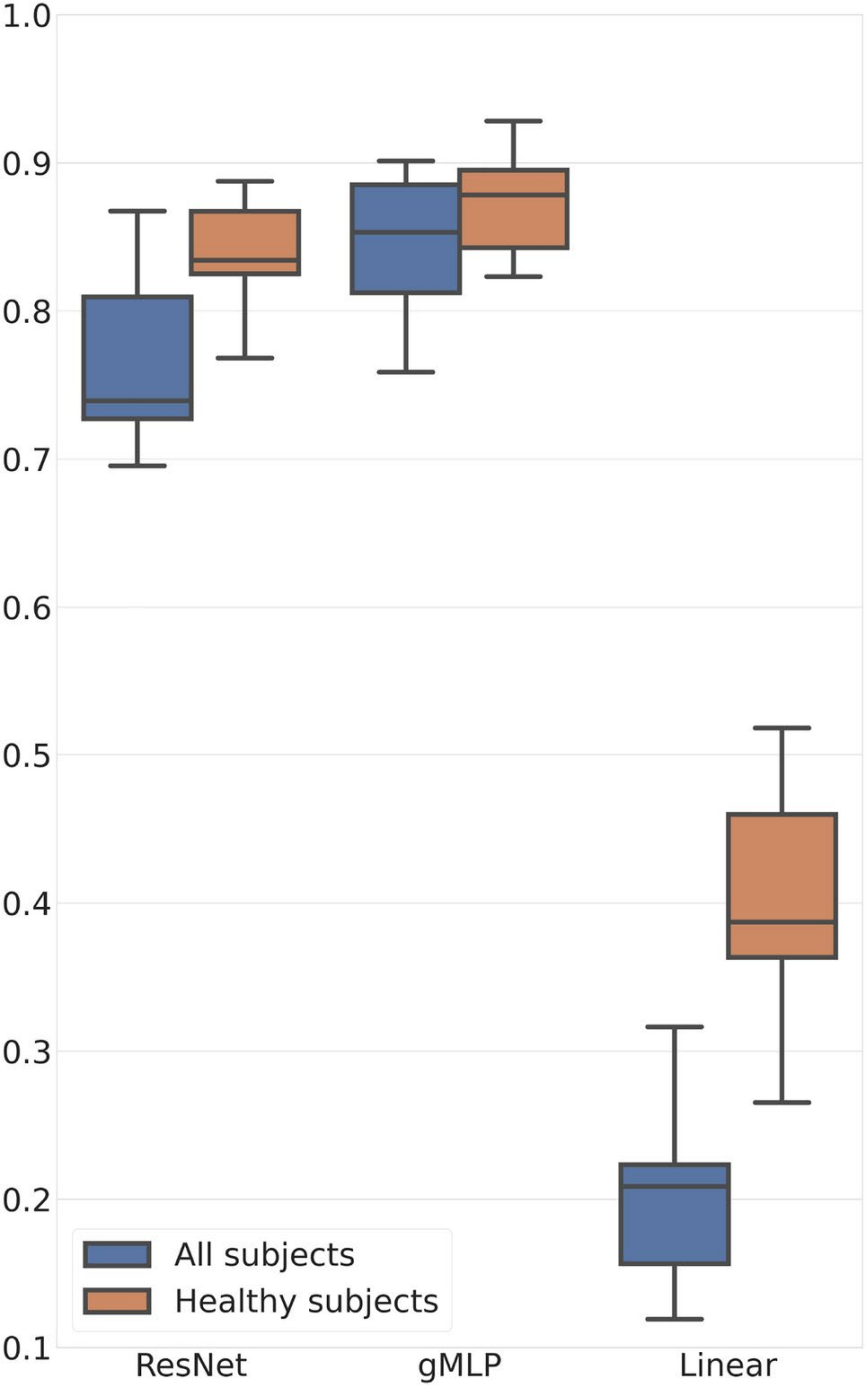
R2 metric box-plots of ResNet, gMLP and Linear Regression models.

Training and testing were performed for two scenarios. First, models were trained for all subjects, including those with toxic maculopathy. Then, only patients without any signs of toxic maculopathy were included in the training and testing process. The results are shown in Fig. 5 and Table 6.

In general, both proposed models (ResNet and gMLP) clearly outperformed the linear model. Furthermore, predictions were consistently more accurate when only subjects without maculopathy were included.

**Table 6:** Evaluation metrics of the models.

Model	Subjects	MSE	R2
Linear Regression	All	1.682	0.204
ResNet	All	1.434	0.767
gMLP^1^	All	1.354	0.842
Linear Regression	Healthy	1.625	0.398
ResNet	Healthy	1.367	0.836
gMLP	Healthy	1.417	0.873

^1^gated Multilayer Perceptron.

## Discussion

In a real-world dataset, we used ML models applied to complete mfERG traces (rather than only the standard amplitudes and implicit times) to detect functional signs of toxic maculopathy. Furthermore, we used such models to predict perimetric sensitivities. Their predictions were better than those of linear models based on the P1 amplitude alone, although they predict non-disease-related variability better than disease-related losses. This suggests that the full traces contain clinically relevant information that is lost when only ring ratios are analyzed. Furthermore, a classification approach worked better than a regression approach in a situation where only a limited number of pathological fields were available.

In the case of toxic maculopathy, three different ML approaches are conceivable. The models can be trained on diagnostic classes as judged by a clinician (classification problem), they can be trained on a continuous variable that is associated with the progression of toxic maculopathy (regression problem), or they can be trained on a prognosis as determined by a long-term follow-up (prognostic problem). The last option would require a very large longitudinal dataset that follows a significant number of patients started on (hydroxy-)chloroquine for at least 5-10 years, so we investigated the first two options.

First, a model can be trained to classify mfERGs to identify toxic maculopathy as judged by a clinician. Choosing a reference (“ground truth”) for training is an essential step in creating diagnostic ML models. The current consensus for the diagnosis of toxic maculopathy is a combination of OCT, visual field testing, and, optionally, mfERG in order to identify early photoreceptor damage^2^. This way, many patients with rheumatic diseases can benefit from an effective and well-tolerated therapy that is not discontinued due to spurious changes^1^. On the other hand, the subgroup of patients who are affected by toxic maculopathy (around 7.5% of all patients for hydroxychloroquine treatment^34^) is protected from severe visual disability (loss of central vision). However, they will suffer some retinal damage with persistent visual field defects. The hope that a more sophisticated analysis of mfERG data may enable the detection of small, reversible functional changes before irreversible photoreceptor damage was neither supported nor contradicted by our study. We did not see clinical differences between patients from the suspect group who were classified as having toxic maculopathy compared with those who were classified as normal (data not shown).

In clinical practice, ring ratios are frequently used for the identification of toxic maculopathy^35^. These are the ratio of the amplitude in the ring under examination $R_n$normalized to that in a reference ring (either R1 or R5)^36^. The values are normally distributed, do not vary significantly with age, and the inter-individual variability was reported to be much smaller in the normal population^37^ compared to amplitudes that are not normalized. However, we used the P1 amplitudes (in ring 2) instead because they had more diagnostic power in the ROC analysis of our data. Furthermore, it is better comparable with the ML analyses based on the full traces because these models rely only on data from one ring.

Habib et al. developed an ML model based on ring ratios, ring variation, and signal strength in a much larger dataset^17^. The study was based on a much larger dataset, but they used a much less sophisticated ML model that was based only on selected parameters. In contrast to our study, the clinical classification was already largely based on ring ratios, which were also an important model input. They reported an F1 score of 69.9%, which is lower than the F1 scores in our models, which are based on the full traces. Although a direct comparison is not possible, this suggests that a model based on the full traces may be superior.

### Regression problem: relationship between perimetry and mfERG

In clinical practice, cut-off values are frequently used to classify patients into discrete groups based on continuous variables. ML models can be trained to either classify patients into groups (classification) or to predict a continuous variable. During the classification process, information is lost. Therefore, a regression model that predicts increasing functional changes with an increasing risk seems better suited than teaching an algorithm to classify patients according to clinical judgment based on already irreversible structural damage.

The macula, a specialized retinal region dominated by cone photoreceptors that is optimized for high spatial resolution and color vision, is characteristic of humans and other primates. All measurements analyzed here are concerned with the macula, but already within this structure, there is a decline in cone photoreceptor packing density with increasing eccentricity^38^. Furthermore, postreceptoral processing of photoreceptor signals changes with eccentricity, and signals are summed up over larger areas.

This leads to a decrease in both perimetric sensitivities and mfERG amplitudes. To mitigate this, mfERG segment areas increase with eccentricity so that amplitudes, a sum of the retinal responses of the whole segment, are approximately constant^39^. In contrast, perimetric sensitivities are not sum responses, and even if larger stimuli are better discernible than smaller ones, sensitivity increases proportionally with size only over a limited range. Thus, perimetric stimuli are not scaled with eccentricity, and we use the amplitude densities, i.e., the amplitudes divided by the area in $deg^2$, for analysis of the mfERG parameters for the classification task.

Sensitivities can be averaged across locations that correspond to one segment in two ways. Either the sensitivity values in decibels are averaged directly, or the de-logarithmized sensitivities values can be averaged and then re-logarithmized. The assumption is that sensitivities in linear scaling have a normal distribution, allowing averaging. In glaucoma, the latter correlates better with structure^40^.

Our data show that ML models that rely on the complete traces, as opposed to only the extracted parameters, predict perimetric sensitivities better than a linear model based on the P1 amplitudes. When comparing perimetry and ERG, a distinction has to be made between physiological variation in normal responses and pathological variation in disease. Normal responses can be correlated between the two modalities due to variations in retinal physiology (for example, cone density) that affect both parameters. Often, these correlations are weak due to the limited variation of these parameters under normal conditions. In comparison, pathological loss, for example, in cone density, may be large compared with physiological variability and thus lead to much closer relationships in both parameters.

Possibly, the models were able to identify variability caused by the eccentricity of the segment under consideration (rings 1 to 3 were used), or it was able to identify age-related changes that affect both mfERG and perimetry. This cannot be determined from the ML models. Even in patients with toxic maculopathy, many segments were not pathologically altered. Using the perimetric defect values, which compare sensitivity to age-correlated normal values, may be better suited for modeling pathological changes than using the sensitivity values themselves.

### Limitations

Our dataset comprises a limited number of patients, and only a small number of these had clear toxic maculopathy. Therefore, it was difficult to train a model for pathological changes, and the external validity may be limited (possibly over-fitting).

Patients with very advanced maculopathy were included in our dataset because exclusion would have further reduced the sample size. We do not believe that this limits the model’s training because these patients also exhibit signs present in earlier diseases. However, validation in an independent dataset that does not include advanced disease is necessary before clinical application because toxic maculopathy needs to be reliably detected early.

All patients were of Caucasian origin. Because Asian patients are known to have more peripheral alteration^41,42^, the model cannot be used in more diverse populations. The retrospective approach is a further limitation, as some data of relevance, including the cumulative dose of the drugs, were not collected in a standardized fashion. Traces with artifacts were discarded by the technician, but we did not check for incorrect positioning of the markers used for extraction of the amplitudes and implicit times.

### Implications for clinicians

Our work aims to provide a universal algorithm for the detection of toxic maculopathy that is ready for clinical application. Such an algorithm needs to be trained on a much larger and more diverse dataset.

However, such a dataset is difficult to obtain even in a large tertiary care center and only few centers have a patient population that is diverse enough to allow application in other centers with different racial background.

Therefore, we experimented with different ML approaches in order to 1) gain insight into how toxic maculopathy affects retinal function, 2) see whether ML can help to identify functional damage in the absence of clear morphological changes, and 3) guide decision making in designing a multi-center approach for developing a clinical application for identification of toxic maculopathy.

We could not identify pre-structural functional changes even with sophisticated ML methods. However, our study demonstrates the potential of applying ML algorithms to mfERG results and shows that complete traces should be provided to the model. This would enable mfERG to be more widely used in settings where no specialist is available. A larger dataset with more affected patients from a more diverse background is necessary to train such a model.

One method that could be applied when including the proposed classificational model in a clinical examination process is human-in-the-loop (HITL)^43^. HITL integrates human expertise with ML to continuously improve the accuracy of the ERG classification model. A medical expert revises and corrects the model’s prediction of the new real clinical cases according to the conventional methods. This feedback is incorporated into the training process, allowing the model to adapt and improve with new data. The iterative feedback loop enhances accuracy, reduces false classifications, and ensures the model remains reliable in a clinical environment.

### Implications for research

Our results show that AI models can be used to investigate relationships between structure and function in retinal disease. Specifically, it shows that it is worthwhile to look at the full ERG curves in order to gain a better understanding which changes are correlated with the improved predictive capabilities of DL models.

We think clinicians, visual scientists, and computer scientists should work together in ways to use all available information and, ideally, learn from these models.

### Conclusions

In our research, we found that in an unbalanced dataset like the one used here, the regression models seemed to predict normal variation better than disease-related variation. This may limit the clinical use of regression models. However, the increase in predictive power by using ML models rather than linear regression is truly impressive. Our data show that using full traces instead of single parameters can significantly enhance diagnostic power in classification tasks. This potential impact of our research is both inspiring and exciting.

## Data Availability

Data are available from the corresponding author upon reasonable request.
